# Global transcriptomic response of *Leptospira interrogans *serovar Copenhageni upon exposure to serum

**DOI:** 10.1186/1471-2180-10-31

**Published:** 2010-01-29

**Authors:** Kanitha Patarakul, Miranda Lo, Ben Adler

**Affiliations:** 1 Faculty of Medicine, Chulalongkorn University, Bangkok 10330, Thailand; 2 ARC Centre of Excellence in Structural and Functional Microbial Genomics, Monash University, Clayton, Victoria 3800, Australia; 3 Department of Microbiology, School of Biomedical Sciences, Monash University, Clayton, Victoria 3800, Australia

## Abstract

**Background:**

Leptospirosis is a zoonosis of worldwide distribution caused by infection with pathogenic serovars of *Leptospira *spp. The most common species, *L. interrogans*, can survive in the environment for lengthy periods of time in between infection of mammalian hosts. Transmission of pathogenic *Leptospira *to humans mostly occurs through abraded skin or mucosal surfaces after direct or indirect contact with infected animals or contaminated soil or water. The spirochete then spreads hematogenously, resulting in multi-organ failure and death in severe cases. Previous DNA microarray studies have identified differentially expressed genes required for adaptation to temperature and osmolarity conditions inside the host compared to those of the environment.

**Results:**

In order to identify genes involved in survival in the early spirochetemic phase of infection, we performed a transcriptional analysis of *L. interrogans *serovar Copenhageni upon exposure to serum in comparison with EMJH medium. One hundred and sixty-eight genes were found to be differentially expressed, of which 55 were up-regulated and 113 were down-regulated. Genes of known or predicted function accounted for 54.5 and 45.1% of up- and down-regulated genes, respectively. Most of the differentially expressed genes were predicted to be involved in transcriptional regulation, translational process, two-component signal transduction systems, cell or membrane biogenesis, and metabolic pathways.

**Conclusions:**

Our study showed global transcriptional changes of pathogenic *Leptospira *upon exposure to serum, representing a specific host environmental cue present in the bloodstream. The presence of serum led to a distinct pattern of gene expression in comparison to those of previous single-stimulus microarray studies on the effect of temperature and osmolarity upshift. The results provide insights into the pathogenesis of leptospirosis during the early bacteremic phase of infection.

## Background

*Leptospira interrogans *is the most common etiologic agent of severe leptospirosis, a zoonotic disease with worldwide distribution [1-3]. Leptospires have been serologically classified based on antigenic determinants into more than 230 serovars. With more recent genetic classification based on DNA relatedness, *Leptospira *has been classified into at least 17 species [1,4-6]. However, no correlation exists between serological and genetic classification. Many species of animals, both domestic and wild, serve as reservoir hosts, resulting in the global spread of the disease. Humans are accidental hosts, with transmission occurring via direct or indirect contact with the urine of infected animals. Pathogenic *Leptospira *can survive for prolonged periods of time in the environment [7]. After gaining entry through skin abrasions or mucous membranes, the spirochete spreads hematogenously to multiple target organs such as the kidneys, liver, and lung, resulting in a wide spectrum of clinical manifestations [1,3]. Therefore, adaptation to various environmental cues outside and within the hosts and the ability to survive in the bloodstream contribute to the ability of leptospires to cause disease.

The responses of leptospires at transcriptional and translational levels to changes in various environmental factors such as temperature, osmolarity, and iron availability have been reported previously [8-13]. Proteins such as Qlp42, Hsp15, LigA, LigB, Sph2, and Lsa21 are up-regulated in response to physiologic temperature or osmolarity [12,14-17]. In contrast, LipL36 is down-regulated at 37°C and during mammalian infection [8,18]. Previous studies demonstrated the *in vivo *expression of several outer membrane proteins, based on the presence of antibodies against these proteins in immune sera or detection of proteins in host tissues infected with pathogenic *Leptospira *[17,19-27]. These proteins, which are expressed *in vivo *or at physiologic conditions, therefore constitute potential virulence-associated factors required for host interaction or survival of *Leptospira *in infected hosts.

DNA microarrays have been used to study genome-wide differential gene expression of bacteria during infection and upon exposure to various stimuli related to *in vivo *conditions [28-32]. Based on available whole-genome sequences of *Leptospira *[33,34], microarray techniques have been utilized to identify a range of genes that are responsive to changes in temperature and/or osmolarity, corresponding to the shift from environmental to physiological conditions [10,11,13]. These microarray studies have usually involved a single stimulus, such as temperature or osmolarity upshift, each resulting in differing expression profiles. However, *L. interrogans *within the mammalian host simultaneously encounters multiple signals that are different from environmental conditions. In the early course of infection, leptospires have to survive and spread in the bloodstream before causing damage to target organs. Blood or serum contains physical, biochemical, and biological properties that are different from those of the *in vitro *environment, such as complement, pH, osmolarity, iron availability, electrolyte concentration, and various serum proteins. Therefore, regulation of gene expression during the spirochetemic phase is the result of integrated and complex stimuli. However, leptospiral genes differentially expressed during the period of bacteremic phase have never been characterized.

In this study, we employed DNA microarray analysis as a tool to identify genes that are differentially expressed in the presence of serum, as these genes may be important in enabling pathogenic *Leptospira *to adapt to and survive in the host environment during the early bacteremic stage of infection. The results were compared to previous microarray data on the responses to changes in temperature and osmolarity [10,11,13].

## Results and discussion

### Serum bactericidal assay

Serum complement plays a crucial role in the innate immune response against bacterial pathogens. To study differential gene expression of *Leptospira *in the presence of serum, we used commercial guinea pig serum with demonstrated complement leptospiricidal activity against *L. biflexa*. Pathogenic leptospires are resistant to the alternative pathway of complement-mediated killing, in contrast to the non-pathogenic species, *L. biflexa *[35-38]. Guinea pigs are susceptible to acute infection with *Leptospira *and have been routinely used as an animal model for leptospirosis [26,39,40]. The same batch of guinea pig serum was used throughout this study to minimize variation between replicate samples.

It is known that pathogenic *Leptospira *may lose virulence after *in vitro *passage [41]. Therefore, serum leptospiricidal activity was tested against different pathogenic serovars available in our laboratory to determine their resistance to complement-mediated killing before use in microarray experiments. The maximum killing (>90%) of non-pathogenic *L. biflexa *serovar Patoc was achieved after incubation with 50% guinea pig serum at 37°C for 30 min (data not shown). Hence, this condition was deemed to be sufficient for pathogenic leptospires to express genes required for survival in serum and was used for subsequent experiments. In this study, low-passage *L. interrogans *serovars Copenhageni and Manilae were shown to be resistant to complement-mediated killing, with 95.5 and 97.3% retention of viability after incubation in serum, respectively, compared to 9% viability of serovar Patoc. However, after incubation with heat-inactivated serum (HIS) the viability of *L. biflexa *was greater than 95%, consistent with the killing effect of serum being due to complement activity. Accordingly, serovar Copenhageni was used in subsequent microarray experiments, since microarray slides were constructed based on the combined complete genome sequences of serovars Lai and Copenhageni available in the database [11].

### Global transcriptomic changes of pathogenic *Leptospira *after serum exposure

Low-passage *L. interrogans *serovar Copenhageni was incubated with 50% guinea pig serum at 37°C for 30 min to simulate *in vivo *conditions encountered upon entry into the host. Comparisons were made with leptospires shifted to 37°C in EMJH medium to exclude the effect of temperature shift, which has previously been reported [10,11].

Overall, 168 genes (4.5% of the genome) were considered to be differentially expressed at a statistically significant level upon serum exposure, i.e. at least 1.5-fold up- or down-regulated with an adjusted *P *value of less than 0.01 as determined by moderated *t *test. Of these, 55 genes (32.7%) were up-regulated and 113 genes (67.3%) were down-regulated (Table 1). Genes of known or predicted function accounted for 54.5% (30 of 55 genes) and 45.1% (51 of 113 genes) of up- and down-regulated genes, respectively.

**Table 1:** Number of leptospiral genes differentially expressed in response to serum compared to EMJH medium

***Genes***	**No. of genes**		
	
	**Up-regulated **(%^a^)	**Down-regulated **(%^a^)	**Total **(%^b^)
Known or predicted function	30 (54.5)	51 (45.1)	**81 (48.2)**
Unknown or poorly characterized function	25 (45.5)	62 (54.9)	**87 (51.8)**

Total	**55**	**113**	**168**

^a ^percentage of genes per total number of genes in up-regulated or down-regulated group

^b ^percentage of genes per total number of differentially expressed genes

**Figure 1:**
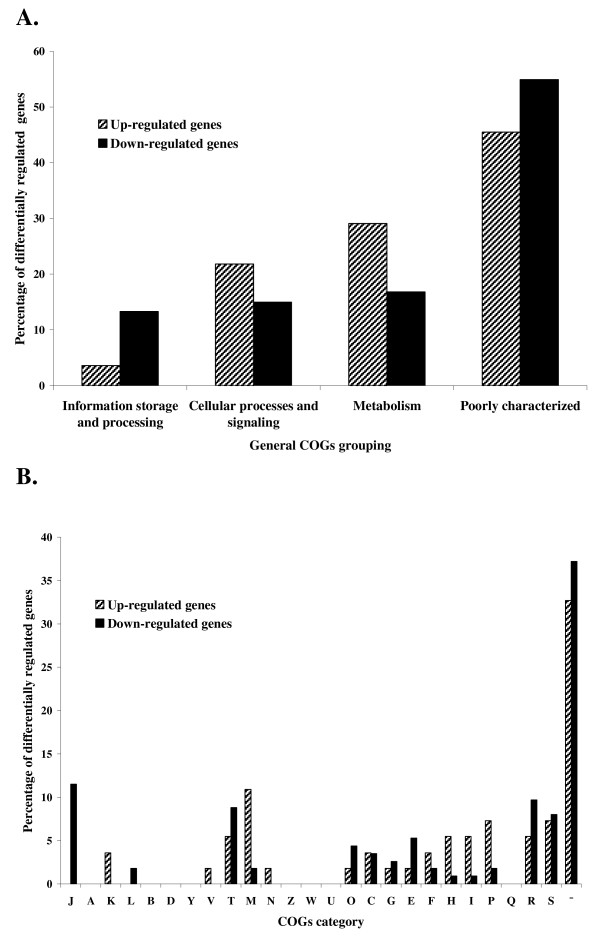
**Percentage of up- and down-regulated genes of *L. interrogans *serovar Copenhageni in response to serum in each general COG grouping (A) and COG category (B)**. The percentage of differentially regulated genes was calculated by dividing number of genes up- and down-regulated in each category by the total number of up- and down-regulated genes, respectively × 100. The COG functional categories are as follows: information storage and processing (includes J, translation; A, RNA processing and modification; K, transcription; L, replication, recombination, and repair; B, chromatin structure and dynamics); cellular processes and signaling (includes D, cell cycle control, cell division, chromosome partitioning; Y, Nuclear structure; V, defense mechanisms; T, signal transduction mechanisms; M, cell wall, membrane, or envelope biogenesis; N, cell motility; Z, cytoskeleton; W, extracellular structures; U, intracellular trafficking, secretion, and vesicular trans- port; O, posttranslational modification, protein turnover, chaperones); metabolism (includes C, energy production and conversion; G, carbohydrate transport and metabolism; E, amino acid transport and metabolism; F, nucleotide transport and metabolism; H, coenzyme transport and metabolism; I, lipid transport and metabolism; P, inorganic ion transport and metabolism; Q, secondary metabolite biosynthesis, transport, and catabolism); poorly characterized (includes R, general function prediction only; S, function unknown; and -, not in COGs).

Differentially expressed genes were classified into functional categories based on clusters of orthologous groups (COGs). The majority of differentially expressed genes were of poorly characterized or unknown function (45.5 and 54.9% of up- and down-regulated genes, respectively) (Figure 1A). In general, of the genes which were serum-inducible, those predicted to be involved in metabolism were overrepresented, followed by the cellular processes and signaling group (Figure 1A). However, down-regulated genes of known or predicted function were similarly distributed in three broad COG categories. Among genes of known or predicted function, the highest proportion of up-regulated genes (10.9%) were those involved in cell wall and membrane biogenesis (COG category M), whereas the largest group of down-regulated genes (11.5%) belonged to COG category J (translation) (Figure 1B).

The most highly up-regulated gene (11.5-fold) was LIC13291, encoding a putative ankyrin repeat protein [Additional file 1]. Ankyrin repeat-containing proteins are ubiquitous proteins that play a role in protein-protein interactions [42-44]. LIC13291 is one of 12 predicted proteins with ankyrin repeat domains in *L. interrogans *[34]. However, protein interactions and partners of ankyrin repeat proteins in *L. interrogans *have not yet been characterized. It is possible that up-regulation of this gene may be crucial for interactions of proteins involved in several functions such as intracellular signaling, nutrient acquisition, and transcriptional regulation to promote survival of *Leptospira *in response to stress conditions encountered in serum.

Interestingly, 11 of 55 (20%) genes that were shown to be up-regulated in our study are unique to *L. interrogans *and are not present in the genome of the saprophytic *L biflexa *[45] [Additional file 1] which is susceptible to complement killing. These up-regulated unique *L. interrogans *genes may encode unique leptospiral virulence factors but their role, if any, in pathogenesis has yet to be determined.

The complete lists of significantly up- and down-regulated genes are shown as [Additional files 1 and 2] respectively. Differentially regulated genes of known or predicted function in each broad COG category (Tables 2 and 3) are discussed below.

**Table 2:** Genes of known or predicted function which were up-regulated in response to serum

*Gene ID^a ^and COG category*	*Gene*	*Fold ratio*	*Description of gene product*	*Temperature effect*^b^	*Osmolarity effect*^c^
**Information storage and processing**					
- Transcription (K)					
LIC11154 (LA2894)		1.70	transcription regulator	-	-
LIC10378 (LA0431)		1.54	transcription regulator, PadR family	-	-

**Cellular process and signaling**					
- defense mechanisms (V)					
LIC12182 (LA1600)		1.58	ATP-binding protein of an ABC transporter complex	-	-
- signal transduction mechanisms (T)					
LIC12979 (LA0599)		2.49	signal transduction protein	-	-
LIC13289 (LA4127)		2.17	sensor histidine kinase of a two- component response regulator	-	↑^d^
LIC10900 (LA3235)		1.72	adenylate/guanylate cyclase	-	-
- cell wall/membrane biogenesis (M)					
LIC11149 (LA2901)		2.75	metallopeptidase	-	-
LIC12151 (LA1632)		2.45	nucleoside-diphosphate sugar epimerase	-	-
LIC10200 (LA0232)		2.17	glycosyltransferase	-	-
LIC10587 (LA3624)		2.07	glycosyltransferase	-	-
LIC11728 (LA2200)		2.01	amidase	-	↑
LIC13469 (LA4326)	*lpxD*	1.65	UDP-3-O-(3-hydroxymyristoyl) glucosamine N-acyltransferase	-	-
- cell motility (N)					
LIC10464 (LA3778)	*ligB*	1.89	LigB lipoprotein	↑	↑
- posttranslational modification, protein turnover, chaperones (O)					
LIC11657 (LA2280)	*fliS*	1.98	endoflagellar biosynthesis chaperone	-	-

**Metabolism**					
**- energy production and conversion (C)**					
**LIC10090 (LA0102)**		1.73	conserved hypothetical protein (FOG:	-	-
			HEAT repeat)		
**LIC20084 (LB107)**		1.71	conserved hypothetical protein related to ferredoxin oxidoreductase	-	-
**- carbohydrate transport and metabolism**					
**(G)**		1.77	permease	-	↑
**LIC20149 (LB187)**					
**- amino acid transport and metabolism (E)**		1.69	acetyltransferase	↑	-
**LIC12184 (LA1598)**					
**- nucleotide transport and metabolism (F)**	*pyrD*	2.01	dihydroorotate dehydrogenase	-	-
**LIC13433 (LA4290)**	*dgt*	1.54	deoxyguanosinetriphosphate	-	-
**LIC11663 (LA2274)**			triphosphohydrolase		
**- coenzyme transport and metabolism (H)**		1.82	pyrimidine reductase	-	↑
**LIC13208 (LA4019)**		1.58	methylase/methyl transferase	-	-
**LIC20082 (LB105)**	*coaE*	1.55	dephospho-CoA kinase	-	-
**LIC13085 (LA3863)**					
**- lipid transport and metabolism (I)**		2.59	fatty acid desaturase	-	-
**LIC20052 (LB068)**	*desA*	2.59	fatty acid desaturase	-	-
**LIC13053 (LA0502)**		2.42	enoyl-CoA hydratase	-	-
**LIC12629 (LA1032)**					
**- inorganic ion transport and metabolism**	*hemO*	2.47	heme oxygenase	-	↑
**(P)**		1.82	Reductase	-	-
**LIC20148 (LB186)**		1.69	cation transport ATPase, possibly copper	↑	-
**LIC13470 (LA4327)**		1.51	Bifunctional permease/carbonic anhydrase	-	-
**LIC12982 (LA0594)**					
**LIC12992 (LA0579)**					

^a^Gene ID is based on predicted ORFs of whole-genome sequence of *L. interrogans *serovar Copenhageni. Gene ID of corresponding serovar Lai is in parenthesis. ORFs of unknown or poorly characterized function were excluded from this table.

^b^Previous microarray data on the effect of overnight 37°C upshift [11] compared to growth at 30°C.

^c^Previous microarray data on the effect of osmolarity upshift [13] compared to EMJH medium.

^d ↑ ^represents up-regulation of gene expression and ↓ represents down-regulation of gene expression.

**Table 3:** Genes of known or predicted function which were down-regulated in response to serum

*Gene ID*^a ^*and COG category*	*Gene*	*Fold ratio*	*Description of gene product*	*Temperature effect*^*b*^	*Osmolarity effect*^*c*^
**Information storage and processing**					
- translation, ribosomal structure and biogenesis (J)					
LIC12111 (LA1677)	*rpsR*	-2.64	30S ribosomal protein S18	-	-
LIC12865 (LA0747)	*rpmC*	-1.91	50S ribosomal protein L29	-	-
LIC12637 (LA1020)	*rpmE*	-1.88	50S ribosomal protein L31	-	-
LIC10750 (LA3423)	*rplA*	-1.82	50S ribosomal protein L1	-	-
LIC12862 (LA0750)	*rplX*	-1.75	50S ribosomal protein L24	-	-
LIC12113 (LA1675)	*rpsF*	-1.70	30S ribosomal protein S6	-	-
LIC12845 (LA0766)	*rplQ*	-1.65	50S ribosomal protein L17	-	-
LIC12774 (LA0851)	*rpmA*	-1.61	50S ribosomal protein L27	-	-
LIC12860 (LA0752)	*rpsN*	-1.59	30S ribosomal protein S14	-	-
LIC12871 (LA0741)	*rplW*	-1.55	50S ribosomal protein L23	-	-
LIC10756 (LA3416)	*rpsG*	-1.54	30S ribosomal protein S7	-	-
LIC10751 (LA3422)	*rplJ*	-1.54	50S ribosomal protein L10	-	-
LIC12855 (LA0757)	*rpmD*	-1.52	50S ribosomal protein L30	-	-
- replication, recombination and repair (L)					
LIC20098 (LB122)		-2.80	XerD related protein (integrase family)	-	↓^d^
LIC12112 (LA1676)	*ssb*	-1.70	single-stranded DNA-binding protein	-	-

**Cellular process and signaling**					
- signal transduction mechanisms (T)					
LIC20012 (LB014)		-2.56	sensor protein of a two-component	-	-
			response regulator		
LIC11201 (LA2829)		-2.16	receiver component of a two-	-	-
			component response regulator		
LIC12762 (LA0866)		-1.97	signal transduction protein	-	↓
LIC12807 (LA0816)		-1.95	receiver component of a two-	↑^d^	-
			component response regulator		
LIC10344 (LA0395)		-1.88	anti-sigma factor antagonist	-	-
LIC13344 (LA4189)		-1.86	anti-sigma regulatory factor (Ser/Thr	-	-
			protein kinase)		
LIC20108 (LB136)		-1.81	anti-sigma factor antagonist	↓	-
LIC20025 (LB031)		-1.77	cyclic nucleotide-binding protein	-	-
LIC11095 (LA2968)		-1.58	adenylate/guanylate cyclase	-	-
LIC12357 (LA1378)		-1.53	membrane GTPase	-	↓
- cell wall/membrane biogenesis (M)					
LIC10271 (LA0312)		-1.66	metallopeptidase, M23/M27 family	↑	-
LIC12621 (LA1044)		-1.54	conserved hypothetical protein	-	-
- posttranslational modification, protein					
turnover, chaperones (O)					
LIC12017 (LA1879)	*clpA*	-2.48	endopeptidase Clp	-	-
LIC12765 (LA0862)	*tpx*	-1.90	peroxiredoxin	↓	↓
LIC13442 (LA4299)	*btuE*	-1.70	glutathione peroxidase	↑	-
LIC20044 (LB058)	*htpG*	-1.68	HSP90	-	-
LIC20093 (LB117)	*bcp*	-1.54	bacterioferritin comigratory protein	-	-

**Metabolism**					
**- energy production and conversion (C)**					
**LIC12002 (LA1897)**	*sdhA*	-1.72	succinate dehydrogenase/fumarate reductase subunit A	-	-
**LIC12476 (LA1222)**	*aceF*	-1.63	dihydrolipoyllysine-residue acetyl		
			transferase and succinyltransferase	-	-
**LIC12217 (LA1553)**	*petE*	-1.62	plastocyanin	-	↓
**LIC12829 (LA0790)**	*gltA*	-1.53	citrate (Si)-synthase	-	-
**- carbohydrate transport and metabolism**					
**(G)**		-1.82	phosphonomutase	-	↓
**LIC12331 (LA1416)**	*mgsA*	-1.72	methylglyoxal synthase	-	-
**LIC12733 (LA0909)**		-1.58	adolase	-	↓
**LIC12233 (LA1532)**					
**- amino acid transport and metabolism (E)**		-2.17	dioxygenase superfamily protein	-	-
**LIC10069 (LA0076)**	*glnK*	-2.17	nitrogen regulatory protein PII	-	-
**LIC10440 (LA3807)**	*csdB*	-1.60	selenocysteine lyase	-	-
**LIC20204 (LB267)**	*speD*	-1.54	adenosylmethionine decarboxylase	-	-
**LIC20239 (LA-SPN3792)**	*gltB*	-1.53	glutamate synthase (NADH)	-	-
**LIC12694 (LA0956)**		-1.52	lactoylglutathione or related lyase	-	-
**LIC10460 (LA3782)**					
**- nucleotide transport and metabolism (F)**		-1.65	purine-nucleoside phosphorylase	-	-
**LIC13399 (LA4248)**	*adk*	-1.55	adenylate kinase	-	-
**LIC12852 (LA0760)**					
**- coenzyme transport and metabolism (H)**	*ubiG*	-1.86	2-polyprenyl-3-methyl-5-		
**LIC10737 (LA3436)**			hydroxy-6-metoxy-1,4-	-	-
			benzoquinol methylase		
**- lipid transport and metabolism (I)**	*ivd*	-1.77		-	-
**LIC10363 (LA0414)**			isovaleryl-CoA dehydrogenase		
**- inorganic ion transport and metabolism**	*amtB*	-3.10		-	-
**(P)**	*kdpA*	-2.09	ammonia permease	↑	-
**LIC10441 (LA3806)**			potassium-transporting ATPase A		
**LIC10990 (LA3112)**			chain		

^a^Gene ID is based on predicted ORFs of whole-genome sequence of *L. interrogans *serovar Copenhageni. Gene ID of corresponding serovar Lai is in parenthesis. ORFs of unknown or poorly characterized function were excluded from this table.

^b^Previous microarray data on the effect of overnight 37°C upshift [11] compared to growth at 30°C.

^c^Previous microarray data on the effect of osmolarity upshift [13] compared to EMJH medium.

^d ↑ ^represents up-regulation of gene expression and ↓ represents down-regulation of gene expression.

#### Information storage and processing

Putative transcriptional regulators including a protein in the PadR family (encoded by LIC10378) were up-regulated in response to serum. PadR has been shown to be a transcriptional repressor of *padA *gene (encoding a phenolic acid decarboxylase) expression in response to phenolic acid stress in *Lactobacillus plantarum *[46,47]. However, the target of the leptospiral PadR homolog remains unknown. In the presence of serum, several subunits of 30S and 50S ribosomal proteins of *Leptospira *were repressed, possibly due to the shift of energy to produce other gene products that are needed for survival in serum. Reduction of ribosomal gene expression has also been found in organisms under various stress conditions such as *Streptococcus pneumoniae *isolated from infected blood [48], *Campylobacter jejuni*, *Staphylococcus aureus*, and *Helicobacter pylori *in response to acid shock [49-51], and *E. coli *under anaerobic and acidic conditions [52] and nitrogen and sulfur starvation [53].

#### Cellular processes and signaling

Serum exposure resulted in both up- and down-regulation of several genes involved in cellular processes and signal transduction. Different genes with the same predicted function, such as putative metallopeptidases (LIC11149 and LIC10271), sensor or receiver proteins of two-component response regulators (LIC20012, LIC11201, LIC12807, LIC12979 and LIC13289), and adenylate/guanylate cyclase (LIC10900 and LIC11095) were found to be regulated in opposite directions. LIC20012, an ortholog of *hklep *encoding a sensor kinase of the Hklep/Rrlep two-component system involved in heme biosynthesis in *L. biflexa *[54], was down-regulated. However, an ortholog of *rrlep *regulator (LIC20013) was not differentially expressed. Moreover, predicted anti-sigma factor (LIC13344) and anti-sigma factor antagonists (LIC10344 and LIC20108) were down-regulated in response to serum. Bacterial anti-sigma factors and anti-sigma factor antagonists are regulatory proteins that control sigma-factor functions in promoter recognition and initiation of RNA polymerase required for cell viability and stress response [55]. Anti-sigma factors bind to and block their cognate sigma factors, while anti-sigma factor antagonists (or anti-anti-sigma factors) form complexes with anti-sigma factors to inhibit their activity. These findings may be attributed to the fact that the genome of *L. interrogans *is predicted to contain at least 79 genes encoding two-component sensor histidine kinase-response regulator proteins, 9 anti-sigma factors, and 19 anti-sigma factor antagonists required for response to various environmental signals [34]. Therefore, complex stimuli in serum encountered by *Leptospira *may simultaneously cause induction and repression of multiple genes involved in signal transduction networks and transcriptional regulation, possibly leading to expression of genes essential for survival under stress conditions and/or pathogenicity of leptospires inside the host. Detailed study of these individual genes is thus clearly warranted.

The gene encoding the LigB lipoprotein was up-regulated in response to serum. LigB interacts with fibronectin and may serve as an adhesin by binding to host extracellular matrix during the early stages of infection [56-58]. However, recent studies with site-directed mutagenesis of *ligB *did not show attenuation of a *ligB *mutant in the hamster model of leptospirosis [59]. This finding does not exclude the role of LigB as a virulence determinant, since previous studies have shown redundancy in extracellular matrix-binding function of leptospiral proteins including a 36-kDa fibronectin-binding protein [60], Lsa24 (also known as LfhA and LenA)[61,62], LigA [16], Len proteins [62], LipL32 [63], and Lsa21 [17]. Our finding is therefore consistent with the hypothesis that LigB plays a role in virulence, but is not essential.

The *lpxD *(LIC13469) gene encoding UDP-3-O-(3-hydroxymyristoyl) glucosamine N-acyltransferase, which catalyzes the third step of lipid A biosynthesis [64], was up-regulated in response to serum. Lipid A modification was previously shown to affect interaction between Gram-negative bacteria and their environment and to confer virulence in some bacteria [65]. In *H. pylori*, *lpxD *was induced after adhesion to AGS gastric cancer cells [66]. Hence, the differential regulation of *lpxD *might allow *L. interrogans *to modify its lipid A, resulting in alteration of the physical properties of the outer membrane in response to changes in environmental conditions. Notably, the *lpxD *is not arranged in an operon in *Leptospira*, and its differential regulation may thus represent a mechanism for varying LPS expression.

Expression of genes encoding proteins predicted to be involved in the heat shock response, such as *clpA *(LIC12017) encoding the ATP-dependent proteolytic subunit of Clp endopeptidase, and *htpG *(LIC20044), encoding the molecular chaperone Hsp90, was down-regulated in response to serum. The result is not surprising since our experiment did not generate a temperature shift between experimental and control samples, i.e. leptospires were incubated in serum and EMJH medium at the same temperature. The expression of these genes may be affected by signals other than temperature. However, further investigation is required to characterize stress signals in serum that cause down-regulation of these genes. Additionally, down-regulation of genes encoding proteins predicted to be involved in oxidative stress, namely *btuE *(LIC13442) encoding glutathione peroxidase, *tpx *(LIC12765) encoding peroxiredoxin, *bcp *(LIC20093) encoding bacterioferritin comigratory protein, and *ubiG *(LIC10737) encoding the last enzyme in ubiquinone biosynthetic pathway [67-69], was observed in serum-incubated leptospires, consistent with an absence of oxidative stress in serum without any host phagocytic or other cells.

#### Metabolism

To survive in the bloodstream, pathogens need to adjust their metabolism in response to nutrient limitations. In our study, several leptospiral genes involved in metabolic processes were up- or down-regulated, depending on available sources of nutrients and energy in serum compared to those in EMJH medium. The gene *hemO *(LIC20148) encoding heme oxygenase was induced 2.47-fold in response to serum. Heme is an essential *in vivo *source of iron required for growth and biological processes, including electron transfer reactions of leptospires during infection [70]. Bacterial heme oxygenases are enzymes that release Fe^2+ ^from heme by cleaving its tetrapyrrole ring in the presence of oxygen [71]. Previous studies have demonstrated that a transposon mutant in *hemO *of pathogenic *Leptospira *could not utilize hemoglobin (Hb) as the sole iron source [72]. In contrast, the growth of this mutant in EMJH medium, which is supplemented with FeSO_4_, was not impaired. Therefore, up-regulation of leptospiral *hemO *is likely to be necessary for iron acquisition during iron limitation conditions in serum. Indeed, HemO is required for disease pathogenesis in hamsters [73]. In addition, real-time RT-PCR was performed on putative genes involved in iron metabolism to examine gene regulation in response to different iron sources, namely Hb and Fe^2+^. The results showed that *hemO *was up-regulated when leptospires were grown in medium supplemented with Hb. Genes encoding TonB-dependent receptors (LIC12898/LA0706, LIC12374/LA1356, LIC11345/LA2641, and LIC10714/LB3468), Fur-like proteins (LIC11006/LA3094, LIC12034/LA1857, LIC11158/LA2887, and LIC20147/LB183), and hemin-binding protein (HbpA encoded by LIC20151/LB191), were not or weakly differentially expressed in response to Hb [72]. Similarly, except for *hemO*, expression of other genes involved in iron acquisition systems [70] was not significantly affected by serum in our study. Notably, one of 12 putative TonB-dependent receptors (LIC11694) [70], was 1.8-fold up-regulated in response to serum (adjusted *P *value = 0.02). It is probable that the expression of genes involved in iron uptake and transport depends on available iron sources in the environment during infection.

Two genes encoding proteins predicted to be involved in nitrogen assimilation, *amtB *(LIC10441), encoding ammonia permease, and *glnK *(LIC10440), encoding nitrogen regulatory protein II (PII), were down-regulated 3.1-fold (the most strongly down-regulated gene in our study) and 2.17-fold, respectively. In bacteria, *glnK *and *amtB *are conserved and co-transcribed as an operon [73]. PII serves as a signal transduction protein for sensing external ammonium availability and nitrogen status of the cell while ammonia permease acts as a channel for ammonium transport [74]. Ammonium is an important source of nitrogen for biosynthesis of amino acids, nucleotides, and biological amines. Expression of the *glnKamtB *operon is generally induced during growth under limited ammonium conditions [73]. Therefore, ammonia appears to be available in sufficient concentrations in serum in comparison to EMJH medium, resulting in down-regulation of the *glnKamtB *operon.

Beta-oxidation of long-chain fatty acids serves as the major mechanism for energy and carbon acquisition by *Leptospira *[33,34,75,76]. The gene encoding a predicted enoyl-CoA hydratase (LIC12629), which catalyzes the second step of fatty acid oxidation [77], was up-regulated in response to serum, but the expression of other genes in the fatty acid oxidation pathway was not altered. However, LIC12629 is located distantly from other genes in the same pathway and is clearly regulated independently. Leptospiral genes predicted to be involved in the tricarboxylic acid (TCA) cycle, namely *gltA *(LIC12829), encoding citrate synthase and *sdhA *(LIC12002), encoding a flavoprotein subunit of succinate dehydrogenase, and *aceF *(LIC12476), encoding a subunit of the pyruvate dehydrogenase complex were down-regulated. The results suggest that acetyl-CoA derived from fatty acid oxidation was less likely to feed into the TCA cycle. In addition, it was previously shown that transcription of several enzymes in the TCA cycle is iron-dependent or regulated by Fur [78]. It is possible that expression of these genes was repressed when leptospires encountered the low-iron milieu in serum. Similar findings were observed in *Yersinia pseudotuberculosis *grown in plasma, resulting in down-regulation of several enzymes of the TCA cycle [79].

The transition of *Leptospira *to serum resulted in up-regulation of *pyrD *(LIC13433), predicted to encode a dihydroorotate dehydrogenase which catalyzes the fourth step in the *de novo *pyrimidine nucleotide biosynthetic pathway [80], possibly due to limited availability of pyrimidine in serum. This finding is consistent with previous reports showing that the scarcity of nucleotide precursors is the key limitation of bacterial growth in blood [81]. Therefore, *de novo *nucleotide biosynthesis may be required for growth of leptospires in serum. However, enzymes involved in *de novo *biosynthesis of purine nucleotides were not induced in our study. Notably, down-regulation of one of the purine salvage enzymes (LIC13399, predicted to encode a purine-nucleoside phosphorylase) was observed. It has been suggested that transcription of genes in purine and pyrimidine biosynthetic pathways is independently regulated [80,81]. In addition, it is possible that differential expression of genes involved in purine biosynthesis was transient and may not show steady-state expression ratios. Therefore, these genes were not detected as differentially expressed. In addition, *coaE *(LIC13085) encoding dephospho-CoA kinase, which catalyzes the final step in coenzyme A biosynthesis [82], was up-regulated in response to serum, consistent with the use of coenzyme A as a key cofactor during serum exposure.

The *kdpFABC *operon is typically induced under conditions of severe K^+ ^limitation or osmotic upshift and repressed during growth in media of high external K^+ ^concentration [83]. The putative *kdpA *(LIC10990) encoding the A chain of potassium-transporting ATPase was down-regulated in response to serum. However, as the level of potassium in EMJH (2.2 mM) is lower than in serum (~5.2 mM) this result is not surprising.

Two leptospiral genes predicted to encode fatty acid desaturases (LIC13053 [*desA*] and LIC20052) were up-regulated in the presence of serum. The unsaturated bonds introduced into fatty acids by these enzymes have been reported to be essential for membrane lipid homeostasis to maintain the fluidity of biological membranes, especially in response to downward temperature shift [84,85]. The ability of *Leptospira *to modulate its membrane lipid using fatty acid desaturases may thus be important for survival in response to environmental stresses encountered in serum.

Bacterial genes of related functions, including enzymes of metabolic pathways, are frequently but not always co-transcribed as a single transcriptional unit. In our study, genes putatively organized in the same operons, such as *hemO *(LIC20148) and LIC20149, and *glnK *and *amtB *genes, were similarly differentially regulated. However, some genes, such as *pyrD *(LIC13433), *kdpA *(LIC10990), and *sdhA *(LIC12002), did not have the same levels of expression as other genes within their putative operons. A possible explanation could be due to transcriptional polarity [86], where the level of expression of distal genes is less than that of promoter-proximal genes. In addition, the expression of the constituent genes in an operon may sometimes be discoordinated at the suboperonic level by the presence of internal promoters, differential translational efficiency, or differential instability of regions of a polycistronic mRNA [87]. This allows a subset of the operon to be separately transcribed as an internal mini-operon in response to different signals. Finally, most predicted operons have not been verified experimentally, and the genes therein can in reality be transcribed independently. The definite answer to these various possibilities must await further investigation.

#### Complement resistance and other virulence determinants

Complement-resistant *L. interrogans *serovar Copenhageni was used in our study. Previous reports demonstrated that complement resistance of pathogenic *Leptospira *is related to factor H-binding, degradation of C3b and C3 convertase, and inhibition of membrane-attack complex deposition [24,38]. Factor H acts as a complement regulator by binding to C3b and displacing Bb from C3 convertases, thereby promoting factor I in cleaving C3b into its inactive form, iC3b [88]. Binding to factor H is one of the mechanisms that bacteria utilize to evade complement killing [89]. LfhA (also known as LenA) and LenB of *L. interrogans *were previously shown to interact with factor H [24,61]. However, in our study, genes encoding these factor H-binding proteins were not significantly up-regulated.

With the exception of LigB, other known or potential virulence determinants that play a role in motility, chemotaxis, colonization or adhesion were not found to be up-regulated after exposure to serum. These include extracellular matrix binding proteins, enzymes capable of host cell membrane degradation such as sphingomyelinase, phosphatase, and hemolysin, as well as surface proteins previously shown to be expressed *in vivo*, including OmpL1, LipL41, LipL32, LipL21, LipL46, Loa22, and Lsa21, [17,19-23,25-27,33,34,90,91]. In addition, recent studies using genome-wide transposon mutagenesis of *L. interrogans *revealed novel virulence genes, LA1641 (or LIC12143) and LA0615 (or LIC12967), which resulted in attenuation in hamsters when the genes were insertionally inactivated [92]. Neither gene was differentially expressed in our experiments.

While it is possible that some virulence-associated proteins may be expressed constitutively or regulated at the post-transcriptional level, transcription of some genes may also be influenced by the presence or absence of components in the EMJH medium. It is also possible that conditions different from those used in our study may be required to stimulate these genes, such as time period of serum exposure, growth phase of leptospires, cell density, mediators released from inflammatory cells, or microbe-host cell interactions. Microarray analyses are also limited in that unstable or short-lived transcripts cannot be accurately measured.

### Comparison with microarray data on effect of temperature and osmolarity changes

**Figure 2:**
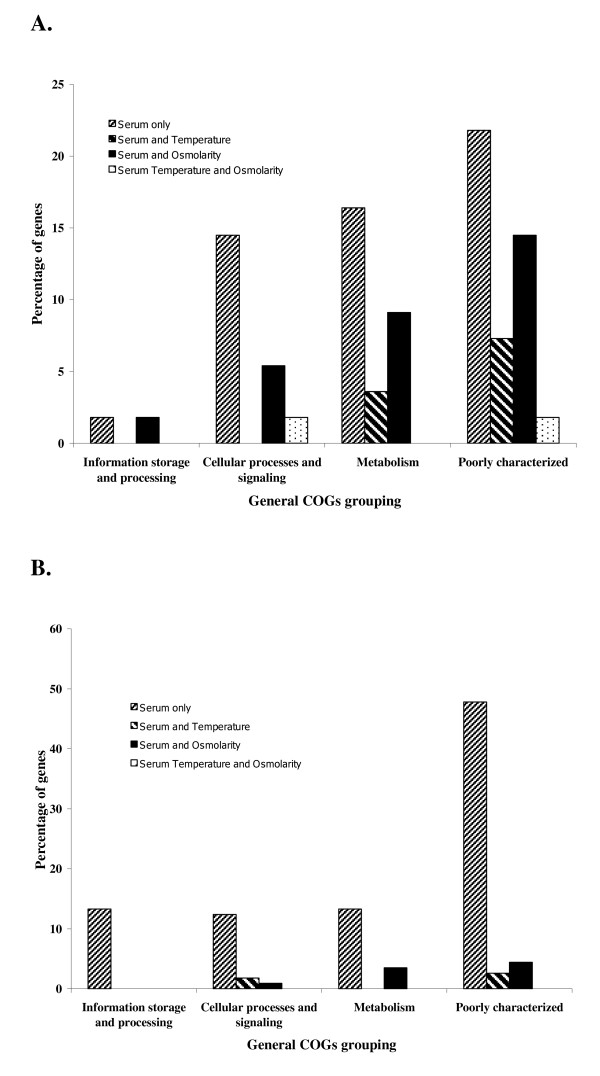
**Percentage of up-regulated (A) and down-regulated (B) genes of *L. interrogans *serovar Copenhageni in response to serum that were differentially expressed due to the effect of: serum only, serum and temperature shift, serum and osmolarity shift, and all three conditions; in each general COG grouping**.

We compared our results with previous microarray data on the effect of temperature and osmolarity changes on leptospiral gene expression [11,13,15]. Due to different criteria applied in these studies, we have re-analysed the previous microarray data using the same statistical criteria (at least 1.5-fold ratio and adjusted *P *value < 0.01). The overnight 37°C upshift vs 30°C dataset [11] was the temperature condition used for comparison. Of the total 168 differentially expressed genes, expression of 36 of 55 (65.5%) up-regulated genes and 94 of 113 (83.2%) down-regulated genes was considered to be serum-specific, i.e. genes that were differentially regulated in response only to serum exposure but not to temperature and/or osmolarity shift [Additional files 1 and 2]. Most leptospiral genes in each general COG grouping that were significantly up-regulated (Figure 2A) or down-regulated (Figure 2B) by serum were not affected by temperature or osmolarity. Hence, serum appeared to generate complex signals that were different from the single-stimulus signal of temperature and osmolarity changes. In the up-regulated group, 20% (11 of 55 genes) were also induced in response to physiological osmolarity shift, whereas only 9.1% (5 of 55 genes) were up-regulated also in response to temperature shift (Table 4). In addition, 3 (2.7%) and 14 (12.4%) of 113 down-regulated genes were also repressed in response to temperature and osmolarity shifts, respectively (Table 4). In other words, the transcriptional profile in response to serum was more similar to that of the response to increased osmolarity rather than to temperature shift. This finding can be attributed to the fact that both the experimental and control samples were incubated at the same temperature and therefore, transcriptional differences due to temperature shift would be excluded. However, differences between our findings and those of previous microarray studies may also be due to variation of experimental conditions between studies, such as the incubation period and cell density.

**Table 4:** Number of leptospiral genes differentially expressed in response to serum compared with the effects of temperature and osmolarity shifts^a^

Serum effect	Temperature effect		Osmolarity effect		Temperature and osmolarity effect	
	
	Up-regulated	Down-regulated	Up-regulated	Down-regulated	Up-regulated	Down-regulated
Up-regulated (%^b^)	5 (9.1)	2 (3.6)	11 (20)	0 (0)	3 (5.6)	0 (0)

Down-regulated (%^c^)	9 (8)	3 (2.7)	2 (1.8)	14 (12.4)	0 (0)	2 (1.8)

^a^compared with previous microarray data of overnight 37°C [11] and osmolarity upshifts [13]

^b^percentage of genes up-regulated in response to serum and/or temperature and/or osmolarity shifts per total number of genes up-regulated in response to serum (55 genes)

^c^percentage of genes down-regulated in response to serum and/or temperature and/or osmolarity shifts per total number of genes down-regulated in response to serum (113 genes)

Interestingly, *ligB *was the only gene of known or predicted function that was up-regulated in response to all three conditions [11,13,15,16]. Therefore, this gene is most likely induced during early bloodstream infection upon exposure to serum and temperature and osmolarity shift. This finding correlates with previous studies showing that anti-LigB IgM was found in more than 95% of patients with acute leptospiral infection [93]. It is therefore intriguing that *ligB *is not essential for acute infection of hamsters or for rat kidney colonization [58]. Interestingly, no gene of known or predicted function was down-regulated by all three signals. In addition, expression of genes encoding proteins known to be temperature regulated, such as LipL36 [8] and Qlp42 [14], was not altered in our study, a finding consistent with previous work on the effect of temperature on these genes [11].

### Validation of microarray data by quantitative RT-PCR

To validate the microarray data, 12 genes were selected for quantitative RT-PCR. Genes encoding flagella subunits, *flaB *and *flaA2 *did not show any transcriptional changes under different temperature or osmolarity conditions and were used for normalization of RT-PCR data in those studies [11,13]. Likewise, *flaB *transcription was not altered by the presence of serum and therefore, *flaB *was used for normalization of RT-PCR data in this study. The correlation coefficient (R^2^) between expression measured by microarray and real-time quantitative PCR was 0.812 [Additional file 3].

## Conclusions

We studied global changes at the transcriptional level of *L. interrogans *serovar Copenhageni in response to serum, thus mimicking the early bacteremic phase of infection. Out of a total of 3,711 ORFs, 168 genes (4.5%) were found to be differentially expressed. To adapt to stress signals in serum, several genes involved in transcriptional regulation, translational process, signal transduction systems, cell or membrane biogenesis, enzymes in various metabolic pathways, and unknown genes were differentially expressed. Serum appeared to be a unique stimulus for leptospires, resulting in a distinct pattern of gene expression compared with genes found to be regulated by only temperature or osmolarity shifts. The only gene of known or predicted function induced by all three conditions was *ligB*. However, many genes previously reported to be virulence associated were not up-regulated in the presence of serum. Expression of these genes may require additional signals that were absent from our study. Alternatively, these genes may be expressed transiently in particular host niches, expressed constitutively or the proteins may be regulated at the translational level. In addition, microarray analyses are also limited in that transcripts which are unstable or have a short half-life are unlikely to be measured accurately. However, our results serve to advance our understanding of genes which may be important in pathogenesis. Genes of unknown function are over represented in the set of genes unique to pathogenic *Leptospira *spp. [45], consistent with the notion that *Leptospira *possesses unique virulence factors. Accordingly, such genes of unknown function that are differentially regulated upon serum exposure warrant further investigation to gain a better insight into their roles in the pathogenesis of leptospirosis.

## Methods

### Bacterial growth and conditions

Pathogenic *L. interrogans *serovar Copenhageni strain L533, and non-pathogenic *L. biflexa *serovar Patoc strain L41 were grown in EMJH broth medium at 30°C under aerobic conditions. Leptospires were grown to exponential phase at an approximate density of 5-8 × 10^8^cells/ml before harvesting by centrifugation at 8000 × g.

### Complement and heat-inactivated sera

Normal guinea pig serum (NGS) (Sigma, St Louis, MO) was obtained lyophilized and stored at -80°C until use. Serum was reconstituted in 1 or 5 ml of sterile ice-cold deionized water according to the manufacturer's instructions. To maintain consistency, the same batch of serum was used throughout. Heat-inactivated serum (HIS) was obtained by incubating NGS at 56°C for 30 min. Sera were freshly prepared before use or stored at -80°C until use. Serum was prewarmed at 37°C for 30 min before incubating with leptospires.

### Serum bactericidal assay

Serum bactericidal assays were performed as described previously with minor modification [38]. Pathogenic leptospires were grown to exponential phase and diluted in liquid EMJH medium to a density of 2 × 10^8^cells/ml before use. 1 × 10^7 ^bacteria were incubated with 50% NGS in a final volume of 100 μl at 37°C for up to 2 h. HIS was used as a control. Samples were taken at different time points and viable spirochetes were enumerated by dark-field microscopy using a Petroff-Hausser counting chamber. The percentage of viable leptospires was calculated by comparison with those incubated with 50% HIS which were considered as 100% viability. The assay was performed in triplicate. The non-pathogenic, complement-sensitive *L. biflexa *serovar Patoc was used in parallel under the same conditions as a control for serum killing.

### Microarray construction

Microarrays were constructed based on a revised annotation of the whole genome sequence of *L. interrogans *serovar Lai strain 56601, with the addition of 45 ORFs unique to *L. interrogans *serovar Copenhageni strain Fiocruz L1-130 as described previously [11].

### Serum exposure and RNA isolation

One hundred ml cultures of *L. interrogans *serovar Copenhageni strain L533 were divided equally between 2 tubes and harvested by centrifugation at 8,000 × g for 20 min at room temperature. The cell pellet in each tube was resuspended in 5 ml of either prewarmed EMJH or prewarmed 50% NGS in EMJH. After incubation at 37°C for 30 min, 0.5 ml of ice-cold killing buffer (50 mM Tris-HCl, pH 7.5, 15 mg/ml sodium azide, 0.6 mg/ml chloramphenicol) was immediately added to each tube before chilling on ice for 5 min. The NGS- and EMJH-treated cells were harvested by centrifugation at 4°C for 15 min and RNA isolated as described previously [11]. The concentration and purity of RNA were measured with a Nanodrop-1000 spectrophotometer (ThermoScientific, Wilmington, DE) and RNA integrity was determined by agarose gel electrophoresis. The lack of DNA contamination in the RNA sample was checked by PCR using 0.5 μg of RNA and primers for *flaB *[Additional file 4].

### Preparation of labeled cDNA probes and microarray hybridization

Each labeled cDNA probe was derived from 2.5 μg of total RNA using the 3DNA Array 900 MPX expression array detection kit (Genisphere, Hatfield, PA) according to the manufacturer's instructions. The comparison between NGS-treated and EMJH-grown samples had 3 biological replicates with a dye swap for each replicate, resulting in 6 arrays. Hybridization was carried out using the 3DNA Array 900 MPX expression array detection kit as per the manufacturer's instructions and as described previously [11].

### Analysis of microarray images and statistical criteria

After hybridization, the microarray slides were immediately scanned with a GMS 418 array scanner (Genetic Microsystems, Woburn, MA). The fluorescent intensities of spots from the Cy3 and Cy5 images were quantitated with ImaGene version 5.1 (Biodiscovery, El Segundo, CA). Spots with poor quality were flagged for elimination from subsequent analysis steps. The web-based program Bioarray Software Environment (BASE) was used for data analysis as described previously [11,13]. Briefly, spot-specific median background intensities were subtracted from spot-specific median signals. Only spots with a corrected intensity of greater than 250 were further analyzed. Data normalization for each array was performed independently using the global median ratio, which scales the intensities such that the median of the ratio between Cy3 and Cy5 channels was 1 and spots within 5% of the lowest and the highest intensities were excluded. Print-tip loess normalization was applied to each array, followed by between-arrays normalization, which scales all replicate arrays such that they had the same median absolute deviation. Direct comparison of gene expression between NGS-treated and EMJH-grown samples was based on moderated *t *test and associated *P *values adjusted for multiple testing by controlling the false discovery rate. Differentially expressed genes were considered to be statistically significant if an absolute relative ratio was greater than 1.5 fold with an adjusted *P *value of less than 0.01. The data discussed in this publication have been deposited in NCBI's Gene Expression Omnibus (Edgar *et al*., 2002) and are accessible through GEO Series accession number GSE17942 http://www.ncbi.nlm.nih.gov/geo/query/acc.cgi?acc=GSE17942.

### Validation of microarray data by qRT-PCR

Twelve differentially expressed genes with varying degrees of up- and down-regulation were selected from the microarray results for qRT-PCR. Primers for real-time RT-PCR were designed using Primer Express software (ABI, Foster City, CA) [Additional file 3]. Each RT reaction mixture contained 5 μg of total RNA, 7.5 μg of random hexamers, 300 units of Superscript III reverse transcriptase (Invitrogen), 1 mM dNTP mix (1 mM each dATP, dGTP, dCTP, and dTTP), 10 mM DTT, and 20 units rRNasin^® ^RNase inhibitor (Promega, Madison, WI). Samples were incubated at 42°C for 2.5 h then at 70°C for 15 min. The synthesized cDNA was diluted 1/50 to 1/100 prior to use in real-time PCR. Real-time PCR reaction mixtures each contained 2.5 μL of cDNA, gene-specific primers at a final concentration of 100 nM each, and 10 μL of SYBR Green PCR master mix (ABI) in a total volume of 20 μL. Real-time PCR was carried out using a Mastercycler ep realplex real-time PCR system (Eppendorf, Hamburg, Germany). Reactions were performed in triplicate. A standard curve for each gene was constructed using known concentrations of *L. interrogans *serovar Copenhageni genomic DNA. The gene encoding flagella subunit B, *flaB*, was used to normalize all data. Melting curve analysis confirmed that all PCRs amplified a single product.

## List of abbreviations

EMJH medium: Ellinghausen-McCullough-Johnson-Harris medium; NGS: normal guinea pig serum; HIS: heat-inactivated guinea pig serum; ORF: open reading frame; qRT-PCR: quantitative reverse transcription polymerase chain reaction.

## Authors' contributions

KP performed the experimental work and statistical analyses under the supervision of ML and BA. ML and BA were involved in microarray design and construction. KP wrote the manuscript with assistance of ML and BA. All authors have read and approved the content of this article.

## Supplementary Material

Additional file 1 **Table S1**. List of genes upregulated in serum, with an adjusted P value of < 0.01. Click here for file

Additional file 2 **Table S2**. List of genes downregulated in serum, with an adjusted P value of < 0.01. Click here for file

Additional file 3 **Figure S1**. Comparison of quantitative RT-PCR and microarray data for twelve genes with varying degrees of up- and down-regulation selected at random. Click here for file

Additional file 4 **Table S3**. Sequences of primers used for PCR and for real-time qRT-PCR to confirm microarray data for some genes. Click here for file
