# Supplementary material for: Global transcriptomic response of Leptospira interrogans serovar Copenhageni upon exposure to serum
Source: BMC Microbiol. 2010 Jan 29;10:31. doi: 10.1186/1471-2180-10-31 (PMC2841595; doi:10.1186/1471-2180-10-31)
Supplement: Additional file 3 — Figure S1. Comparison of quantitative RT-PCR and microarray data for twelve genes with varying degrees of up- and down-regulation selected at random. [file 1471-2180-10-31-S3.DOC]

**Table S3.** Primers used for PCR and real-time qRT-PCR in this study

| **Gene or ORF** | **Forward primer** | **Reverse primer** |
| --- | --- | --- |
| *flaB* (LIC11890) | GAGAGAAACACCGAAGACGG | TGAATAGCAAGAACCCGGAT |
| LIC10244 | GGATTTGGCGGAAGCATTG | TCAACTCTTTCATCAACCAGCG |
| LIC11885 | AATCTCCACTGCGGTAGACAATC | TTGCAGGAAAAACGGGAGC |
| LIC12017 | TTGCAGATCCAAATCGTCCC | GTCAAACAAGAATTCGGCGAG |
| LIC12032 | GCCGCATTTTCCCCTTCTAA | CGATACCTATGAGCATCCGGA |
| LIC13291 | ATGATCGCAGCAAAGGAAGG | ATCAACGCTGTATGCCCGTC |
| *ligB* (LIC10464) | GAATATTACGGATTCGACATACATCG | CCTTGGATGGTTACAACGGATT |
| LIC12982 | TTCGCGCTCTTAGAAATGGG | GCGGCAGACGTTCCTAAAAC |
| LIC10441 | AGATTTTGCTGGTGGAACCG | TCAGAGCGGGTCCTTCTCCT |
| LIC12090 | GGCGCCGAAAAACATATCAA | TCGTTGTTTACACCCATGACG |
| LIC12339 | ACAGTGGCTACCCCTGGAAA | CCCCAACCTGAATTCCAAGA |
| LIC10665 | ATTTCAATCGCGGACCAGAG | GATTGCGCCGAGCAGTTT |
| LIC20098 | AACAACCCGAATGCGATCAC | CCGAAATAAACCAGACCACACA |
