# Supplementary material for: Global transcriptomic response of Leptospira interrogans serovar Copenhageni upon exposure to serum
Source: BMC Microbiol. 2010 Jan 29;10:31. doi: 10.1186/1471-2180-10-31 (PMC2841595; doi:10.1186/1471-2180-10-31)
Supplement: Additional file 4 — Table S3. Sequences of primers used for PCR and for real-time qRT-PCR to confirm microarray data for some genes. [file 1471-2180-10-31-S4.DOC]

**Figure S1.** Comparison of real-time RT-PCR and microarray data. Twelve genes with varying degrees of up- and down-regulation were selected at random for real-time RT-PCR analysis. The correlation coefficient (R^2^) was 0.812.
